# *t*-3-Ethyl-*r*-2,*c*-6-bis­(4-methoxy­phen­yl)-1-nitro­sopiperidin-4-one

**DOI:** 10.1107/S1600536809024878

**Published:** 2009-07-04

**Authors:** T. Kavitha, M. Thenmozhi, S. Ponnuswamy, P. Sakthivel, M. N. Ponnuswamy

**Affiliations:** a Centre of Advanced Study in Crystallography and Biophysics, University of Madras, Guindy Campus, Chennai 600 025, India; b Department of Chemistry, Government Arts College (Autonomous), Coimbatore 641 018, Tamilnadu, India

## Abstract

In the title mol­ecule, C_21_H_24_N_2_O_4_, the piperidine ring adopts a distorted boat conformation with the ethyl substituent in the axial position. The dihedral angle between the two benzene rings is 70.25 (9)°. An intra­molecular C—H⋯O inter­action is observed. In the crystal, mol­ecules are linked into a chain along the *c* axis by C—H⋯O hydrogen bonds and the chains are linked *via* weak C—H⋯π inter­actions.

## Related literature

For general background to 4-piperidones, see: Wang *et al.* (1992 ▶); Grishina *et al.* (1994 ▶). For ring conformational analysis, see: Cremer & Pople (1975 ▶).
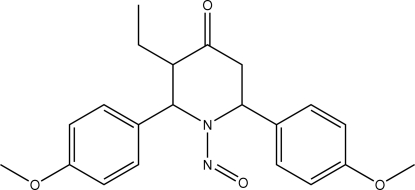


## Experimental

### 

#### Crystal data

C_21_H_24_N_2_O_4_
*M* *_r_* = 368.42
Orthorhombic, 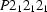

*a* = 7.2742 (4) Å
*b* = 15.8459 (7) Å
*c* = 16.4800 (7) Å
*V* = 1899.59 (16) Å^3^
*Z* = 4
Mo *K*α radiation
μ = 0.09 mm^−1^
*T* = 293 K
0.25 × 0.20 × 0.20 mm

#### Data collection

Bruker Kappa APEXII CCD area-detector diffractometer
Absorption correction: multi-scan (*SADABS*; Sheldrick, 2001 ▶) *T*_min_ = 0.978, *T*_max_ = 0.982
15051 measured reflections
3334 independent reflections
2550 reflections with *I* > 2σ(*I*)
*R*_int_ = 0.026

#### Refinement

*R*[*F* ^2^ > 2σ(*F* ^2^)] = 0.040
*wR*(*F* ^2^) = 0.107
*S* = 1.02
3334 reflections
244 parameters
H-atom parameters constrained
Δρ_max_ = 0.20 e Å^−3^
Δρ_min_ = −0.17 e Å^−3^

### 

Data collection: *APEX2* (Bruker, 2004 ▶); cell refinement: *SAINT* (Bruker, 2004 ▶); data reduction: *SAINT*; program(s) used to solve structure: *SHELXS97* (Sheldrick, 2008 ▶); program(s) used to refine structure: *SHELXL97* (Sheldrick, 2008 ▶); molecular graphics: *ORTEP-3* (Farrugia, 1997 ▶); software used to prepare material for publication: *SHELXL97* and *PLATON* (Spek, 2009 ▶).

## Supplementary Material

Crystal structure: contains datablocks I, global. DOI: 10.1107/S1600536809024878/ci2810sup1.cif

Structure factors: contains datablocks I. DOI: 10.1107/S1600536809024878/ci2810Isup2.hkl

Additional supplementary materials:  crystallographic information; 3D view; checkCIF report

## Figures and Tables

**Table 1:** Hydrogen-bond geometry (Å, °)

*D*—H⋯*A*	*D*—H	H⋯*A*	*D*⋯*A*	*D*—H⋯*A*
C15—H15*C*⋯O1	0.96	2.56	3.163 (4)	121
C18—H18⋯O1^i^	0.93	2.56	3.472 (3)	167
C23—H23*B*⋯*Cg*1^ii^	0.96	2.89	3.718 (2)	144

Symmetry codes: (i) 
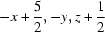
; (ii) 
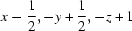
. *Cg*1 is the centroid of the C16–C21 ring.

**Fig. 1.**
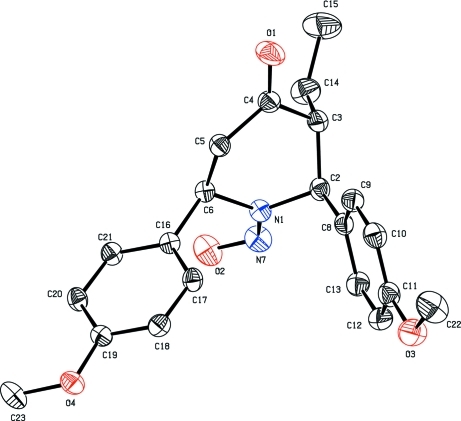
Molecular structure of the title compound. Displacement ellipsoids are drawn at the 30% probability level. H atoms have been omitted for clarity.

**Fig. 2.**
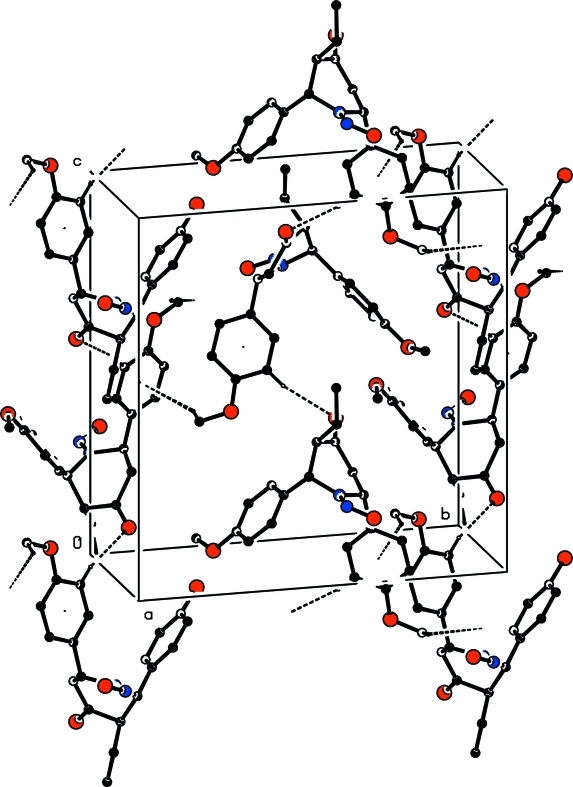
The molecular packing of the title compound, viewed approximately along the a axis. Dashed lines indicate hydrogen bonds. H atoms not involved in hydrogen bonding have been omitted.

## supplementary crystallographic information

### Comment

Piperidine derivatives, namely 4-piperidones, are synthetic intermediates in the preparation of various alkaloids and pharmaceutical products (Wang *et al.*, 1992; Grishina *et al.*, 1994).

The piperidine ring adopts a distorted boat conformation, with the ethyl substituent at C3 position in the axial orientation. The puckering parameters for the piperidine ring are q2 = 0.591 (2) Å, q3 = 0.097 (2) Å, Q_T_ = 0.599 (2) Å and φ2 = 73.2 (2)° (Cremer & Pople, 1975). The dihedral angle between the two benzene rings is 70.25 (9)°. The sum of the bond angles around N1 (359°) indicates *sp*^2^ hybridization. An intramolecular C—H···O interaction is observed.

The crystal structure is stabilized by intermolecular C—H···O hydrogen bonds (Table 1) which link the molecules into a chain along the *c* axis. The chains are linked via C—H···π interactions involving the C16-C21 ring.

### Experimental

To a solution of *t*-3-ethyl-*r*-2,*c*-6-bis(4-methoxyphenyl)piperidin-4-one (1.69 g, 5 mmol) in chloroform (10 ml) was added conc. HCl (1.5 ml) and water (1.5 ml) and while stirring, solid NaNO_2_ (0.84 g,12 mmol) was added in portions during 0.5 h. The solution was stirred at room temperature for another 0.5 h. The organic layer was washed with water, saturated aqueous NaHCO_3_ and dried over anhydrous Na_2_SO_4_. The resulting solution was concentrated and the residue was crystallized from ethanol.

### Refinement

H atoms were positioned geometrically (C-H = 0.93–0.98 Å) and allowed to ride on their parent atoms, with *U*_iso_(H) = 1.2-1.5*U*_eq_(C). In the absence of significant anomalous scattering effects, the Fridel pairs were averaged.

### Crystal data

**Table:**

C_21_H_24_N_2_O_4_	*F*(000) = 784
*M**_r_* = 368.42	*D*_x_ = 1.288 Mg m^−^^3^
Orthorhombic, *P*2_1_2_1_2_1_	Mo *K*α radiation, λ = 0.71073 Å
Hall symbol: P 2ac 2ab	Cell parameters from 3334 reflections
*a* = 7.2742 (4) Å	θ = 2.5–31.8°
*b* = 15.8459 (7) Å	µ = 0.09 mm^−^^1^
*c* = 16.4800 (7) Å	*T* = 293 K
*V* = 1899.59 (16) Å^3^	Block, colourless
*Z* = 4	0.25 × 0.20 × 0.20 mm

### Data collection

**Table:**

Bruker Kappa APEXII CCD area-detector diffractometer	3334 independent reflections
Radiation source: fine-focus sealed tube	2550 reflections with *I* > 2σ(*I*)
graphite	*R*_int_ = 0.026
ω scans	θ_max_ = 31.8°, θ_min_ = 2.5°
Absorption correction: multi-scan (*SADABS*; Sheldrick, 2001)	*h* = −10→10
*T*_min_ = 0.978, *T*_max_ = 0.982	*k* = −23→16
15051 measured reflections	*l* = −15→24

### Refinement

**Table:**

Refinement on *F*^2^	Primary atom site location: structure-invariant direct methods
Least-squares matrix: full	Secondary atom site location: difference Fourier map
*R*[*F*^2^ > 2σ(*F*^2^)] = 0.040	Hydrogen site location: inferred from neighbouring sites
*wR*(*F*^2^) = 0.107	H-atom parameters constrained
*S* = 1.02	*w* = 1/[σ^2^(*F*_o_^2^) + (0.0555*P*)^2^ + 0.1347*P*] where *P* = (*F*_o_^2^ + 2*F*_c_^2^)/3
3334 reflections	(Δ/σ)_max_ = 0.001
244 parameters	Δρ_max_ = 0.20 e Å^−^^3^
0 restraints	Δρ_min_ = −0.16 e Å^−^^3^

### Special details

**Table:**

Geometry. All e.s.d.'s (except the e.s.d. in the dihedral angle between two l.s. planes) are estimated using the full covariance matrix. The cell e.s.d.'s are taken into account individually in the estimation of e.s.d.'s in distances, angles and torsion angles; correlations between e.s.d.'s in cell parameters are only used when they are defined by crystal symmetry. An approximate (isotropic) treatment of cell e.s.d.'s is used for estimating e.s.d.'s involving l.s. planes.
Refinement. Refinement of *F*^2^ against ALL reflections. The weighted *R*-factor *wR* and goodness of fit *S* are based on *F*^2^, conventional *R*-factors *R* are based on *F*, with *F* set to zero for negative *F*^2^. The threshold expression of *F*^2^ > σ(*F*^2^) is used only for calculating *R*-factors(gt) *etc*. and is not relevant to the choice of reflections for refinement. *R*-factors based on *F*^2^ are statistically about twice as large as those based on *F*, and *R*- factors based on ALL data will be even larger.

### Fractional atomic coordinates and isotropic or equivalent isotropic displacement parameters (Å^2^)

**Table:**

	*x*	*y*	*z*	*U*_iso_*/*U*_eq_	
O1	1.2631 (2)	0.06968 (12)	0.09708 (10)	0.0722 (5)	
O2	0.56881 (19)	0.08201 (12)	0.27581 (11)	0.0648 (4)	
O3	1.2829 (2)	−0.26039 (11)	0.42094 (11)	0.0677 (5)	
O4	0.96286 (19)	0.17870 (9)	0.59490 (8)	0.0480 (3)	
N1	0.82220 (18)	0.02169 (10)	0.24151 (9)	0.0379 (3)	
C2	0.9060 (2)	−0.04979 (11)	0.19832 (11)	0.0394 (4)	
H2	0.8044	−0.0852	0.1792	0.047*	
C3	1.0038 (3)	−0.01651 (12)	0.12220 (12)	0.0431 (4)	
H3	1.0693	−0.0633	0.0962	0.052*	
C4	1.1403 (3)	0.05176 (12)	0.14271 (12)	0.0439 (4)	
C5	1.1176 (2)	0.09628 (12)	0.22290 (12)	0.0406 (4)	
H5A	1.1624	0.1536	0.2166	0.049*	
H5B	1.1967	0.0686	0.2621	0.049*	
C6	0.9234 (2)	0.10068 (11)	0.25901 (11)	0.0361 (4)	
H6	0.8583	0.1469	0.2319	0.043*	
N7	0.6402 (2)	0.01792 (13)	0.24877 (11)	0.0523 (4)	
C8	1.0192 (2)	−0.10403 (10)	0.25549 (12)	0.0383 (4)	
C9	1.2088 (3)	−0.11160 (12)	0.25259 (13)	0.0452 (4)	
H9	1.2747	−0.0822	0.2134	0.054*	
C10	1.3015 (3)	−0.16248 (13)	0.30744 (13)	0.0496 (5)	
H10	1.4290	−0.1664	0.3051	0.060*	
C11	1.2061 (3)	−0.20718 (12)	0.36542 (13)	0.0484 (5)	
C12	1.0155 (3)	−0.20026 (13)	0.36894 (14)	0.0516 (5)	
H12	0.9496	−0.2303	0.4077	0.062*	
C13	0.9252 (3)	−0.14900 (12)	0.31510 (13)	0.0473 (5)	
H13	0.7980	−0.1441	0.3184	0.057*	
C16	0.9304 (2)	0.12116 (10)	0.34877 (11)	0.0351 (4)	
C17	1.0182 (2)	0.06863 (11)	0.40442 (12)	0.0407 (4)	
H17	1.0712	0.0186	0.3865	0.049*	
C18	1.0279 (2)	0.08954 (12)	0.48551 (11)	0.0411 (4)	
H18	1.0871	0.0537	0.5218	0.049*	
C19	0.9491 (2)	0.16428 (11)	0.51316 (11)	0.0373 (4)	
C20	0.8647 (3)	0.21793 (12)	0.45857 (11)	0.0415 (4)	
H20	0.8142	0.2686	0.4762	0.050*	
C21	0.8557 (2)	0.19574 (11)	0.37750 (12)	0.0402 (4)	
H21	0.7977	0.2320	0.3412	0.048*	
C22	1.4718 (4)	−0.28172 (19)	0.41023 (18)	0.0810 (8)	
H22A	1.5095	−0.3196	0.4525	0.122*	
H22B	1.5451	−0.2314	0.4125	0.122*	
H22C	1.4881	−0.3085	0.3585	0.122*	
C23	0.8815 (3)	0.25378 (15)	0.62544 (14)	0.0602 (6)	
H23A	0.8998	0.2567	0.6831	0.090*	
H23B	0.7521	0.2535	0.6139	0.090*	
H23C	0.9375	0.3019	0.6000	0.090*	
C14	0.8639 (4)	0.02004 (17)	0.06142 (14)	0.0644 (6)	
H14A	0.7562	−0.0160	0.0604	0.077*	
H14B	0.8255	0.0753	0.0802	0.077*	
C15	0.9381 (5)	0.0281 (2)	−0.02394 (16)	0.0977 (10)	
H15A	0.8446	0.0512	−0.0586	0.147*	
H15B	0.9737	−0.0265	−0.0436	0.147*	
H15C	1.0430	0.0649	−0.0238	0.147*	

### Atomic displacement parameters (Å^2^)

**Table:**

	*U*^11^	*U*^22^	*U*^33^	*U*^12^	*U*^13^	*U*^23^
O1	0.0685 (10)	0.0868 (12)	0.0614 (10)	−0.0296 (9)	0.0256 (9)	−0.0182 (9)
O2	0.0365 (7)	0.0822 (10)	0.0758 (11)	0.0125 (7)	−0.0027 (7)	−0.0142 (10)
O3	0.0698 (10)	0.0673 (10)	0.0660 (10)	0.0246 (8)	0.0030 (9)	0.0130 (9)
O4	0.0508 (8)	0.0563 (8)	0.0369 (7)	0.0020 (6)	−0.0037 (6)	−0.0045 (6)
N1	0.0286 (6)	0.0435 (7)	0.0416 (8)	−0.0018 (6)	−0.0024 (6)	−0.0043 (7)
C2	0.0366 (8)	0.0379 (8)	0.0436 (10)	−0.0058 (7)	−0.0018 (7)	−0.0076 (8)
C3	0.0489 (10)	0.0419 (8)	0.0386 (10)	−0.0050 (8)	0.0007 (8)	−0.0061 (8)
C4	0.0426 (9)	0.0461 (9)	0.0429 (10)	−0.0062 (8)	0.0033 (8)	−0.0022 (9)
C5	0.0368 (8)	0.0413 (9)	0.0437 (10)	−0.0063 (7)	0.0010 (7)	−0.0054 (8)
C6	0.0340 (7)	0.0357 (8)	0.0384 (10)	0.0006 (6)	−0.0017 (7)	−0.0023 (8)
N7	0.0310 (7)	0.0675 (11)	0.0585 (11)	−0.0007 (8)	−0.0035 (7)	−0.0043 (9)
C8	0.0381 (8)	0.0340 (8)	0.0427 (10)	−0.0032 (6)	0.0031 (7)	−0.0075 (8)
C9	0.0404 (9)	0.0447 (10)	0.0505 (11)	−0.0009 (7)	0.0062 (8)	−0.0027 (9)
C10	0.0394 (9)	0.0521 (10)	0.0574 (13)	0.0091 (8)	0.0038 (9)	−0.0057 (10)
C11	0.0527 (11)	0.0431 (9)	0.0495 (12)	0.0111 (8)	−0.0008 (9)	−0.0020 (10)
C12	0.0532 (11)	0.0494 (10)	0.0521 (12)	−0.0015 (9)	0.0085 (10)	0.0030 (10)
C13	0.0387 (9)	0.0458 (10)	0.0573 (13)	−0.0034 (8)	0.0039 (9)	−0.0017 (10)
C16	0.0296 (7)	0.0377 (8)	0.0381 (9)	0.0008 (6)	−0.0025 (6)	−0.0011 (8)
C17	0.0403 (9)	0.0364 (8)	0.0455 (10)	0.0067 (7)	−0.0038 (8)	−0.0025 (8)
C18	0.0395 (9)	0.0412 (9)	0.0426 (10)	0.0027 (7)	−0.0063 (7)	0.0049 (8)
C19	0.0309 (7)	0.0441 (9)	0.0369 (9)	−0.0032 (7)	−0.0008 (7)	−0.0033 (8)
C20	0.0413 (9)	0.0393 (8)	0.0439 (10)	0.0085 (7)	−0.0012 (8)	−0.0052 (8)
C21	0.0381 (9)	0.0403 (8)	0.0422 (10)	0.0093 (7)	−0.0046 (7)	0.0011 (8)
C22	0.0746 (17)	0.0949 (19)	0.0736 (17)	0.0407 (15)	−0.0056 (14)	0.0020 (16)
C23	0.0586 (12)	0.0756 (14)	0.0465 (12)	0.0077 (11)	0.0004 (10)	−0.0187 (12)
C14	0.0659 (13)	0.0755 (15)	0.0517 (13)	−0.0138 (12)	−0.0142 (11)	0.0046 (12)
C15	0.117 (3)	0.129 (3)	0.0469 (15)	−0.005 (2)	−0.0102 (16)	0.0193 (18)

### Geometric parameters (Å, °)

**Table:**

O1—C4	1.202 (2)	C11—C12	1.392 (3)
O2—N7	1.225 (2)	C12—C13	1.370 (3)
O3—C11	1.364 (3)	C12—H12	0.93
O3—C22	1.426 (3)	C13—H13	0.93
O4—C19	1.370 (2)	C16—C21	1.384 (2)
O4—C23	1.421 (2)	C16—C17	1.394 (2)
N1—N7	1.331 (2)	C17—C18	1.379 (3)
N1—C2	1.470 (2)	C17—H17	0.93
N1—C6	1.481 (2)	C18—C19	1.392 (3)
C2—C8	1.518 (3)	C18—H18	0.93
C2—C3	1.536 (3)	C19—C20	1.382 (3)
C2—H2	0.98	C20—C21	1.383 (3)
C3—C4	1.506 (3)	C20—H20	0.93
C3—C14	1.541 (3)	C21—H21	0.93
C3—H3	0.98	C22—H22A	0.96
C4—C5	1.507 (3)	C22—H22B	0.96
C5—C6	1.535 (2)	C22—H22C	0.96
C5—H5A	0.97	C23—H23A	0.96
C5—H5B	0.97	C23—H23B	0.96
C6—C16	1.515 (3)	C23—H23C	0.96
C6—H6	0.98	C14—C15	1.512 (4)
C8—C9	1.385 (3)	C14—H14A	0.97
C8—C13	1.393 (3)	C14—H14B	0.97
C9—C10	1.386 (3)	C15—H15A	0.96
C9—H9	0.93	C15—H15B	0.96
C10—C11	1.377 (3)	C15—H15C	0.96
C10—H10	0.93		
			
C11—O3—C22	117.3 (2)	C11—C12—H12	120.1
C19—O4—C23	117.23 (16)	C12—C13—C8	121.65 (18)
N7—N1—C2	114.93 (15)	C12—C13—H13	119.2
N7—N1—C6	121.01 (16)	C8—C13—H13	119.2
C2—N1—C6	122.65 (13)	C21—C16—C17	117.70 (17)
N1—C2—C8	111.15 (14)	C21—C16—C6	120.23 (15)
N1—C2—C3	108.85 (14)	C17—C16—C6	122.00 (15)
C8—C2—C3	116.74 (15)	C18—C17—C16	121.19 (16)
N1—C2—H2	106.5	C18—C17—H17	119.4
C8—C2—H2	106.5	C16—C17—H17	119.4
C3—C2—H2	106.5	C17—C18—C19	120.04 (17)
C4—C3—C2	111.62 (15)	C17—C18—H18	120.0
C4—C3—C14	108.13 (17)	C19—C18—H18	120.0
C2—C3—C14	110.74 (17)	O4—C19—C20	124.75 (17)
C4—C3—H3	108.8	O4—C19—C18	115.70 (16)
C2—C3—H3	108.8	C20—C19—C18	119.55 (17)
C14—C3—H3	108.8	C19—C20—C21	119.58 (16)
O1—C4—C3	121.27 (18)	C19—C20—H20	120.2
O1—C4—C5	121.28 (17)	C21—C20—H20	120.2
C3—C4—C5	117.44 (16)	C20—C21—C16	121.92 (17)
C4—C5—C6	117.51 (15)	C20—C21—H21	119.0
C4—C5—H5A	107.9	C16—C21—H21	119.0
C6—C5—H5A	107.9	O3—C22—H22A	109.5
C4—C5—H5B	107.9	O3—C22—H22B	109.5
C6—C5—H5B	107.9	H22A—C22—H22B	109.5
H5A—C5—H5B	107.2	O3—C22—H22C	109.5
N1—C6—C16	112.83 (15)	H22A—C22—H22C	109.5
N1—C6—C5	110.11 (14)	H22B—C22—H22C	109.5
C16—C6—C5	110.94 (14)	O4—C23—H23A	109.5
N1—C6—H6	107.6	O4—C23—H23B	109.5
C16—C6—H6	107.6	H23A—C23—H23B	109.5
C5—C6—H6	107.6	O4—C23—H23C	109.5
O2—N7—N1	114.66 (17)	H23A—C23—H23C	109.5
C9—C8—C13	117.96 (18)	H23B—C23—H23C	109.5
C9—C8—C2	124.60 (17)	C15—C14—C3	113.7 (2)
C13—C8—C2	117.44 (16)	C15—C14—H14A	108.8
C8—C9—C10	120.83 (19)	C3—C14—H14A	108.8
C8—C9—H9	119.6	C15—C14—H14B	108.8
C10—C9—H9	119.6	C3—C14—H14B	108.8
C11—C10—C9	120.40 (18)	H14A—C14—H14B	107.7
C11—C10—H10	119.8	C14—C15—H15A	109.5
C9—C10—H10	119.8	C14—C15—H15B	109.5
O3—C11—C10	125.24 (18)	H15A—C15—H15B	109.5
O3—C11—C12	115.4 (2)	C14—C15—H15C	109.5
C10—C11—C12	119.37 (19)	H15A—C15—H15C	109.5
C13—C12—C11	119.8 (2)	H15B—C15—H15C	109.5
C13—C12—H12	120.1		
			
N7—N1—C2—C8	−111.25 (18)	C8—C9—C10—C11	0.7 (3)
C6—N1—C2—C8	82.2 (2)	C22—O3—C11—C10	−10.4 (3)
N7—N1—C2—C3	118.82 (18)	C22—O3—C11—C12	168.6 (2)
C6—N1—C2—C3	−47.7 (2)	C9—C10—C11—O3	178.42 (19)
N1—C2—C3—C4	54.76 (19)	C9—C10—C11—C12	−0.6 (3)
C8—C2—C3—C4	−72.04 (19)	O3—C11—C12—C13	−179.33 (18)
N1—C2—C3—C14	−65.78 (19)	C10—C11—C12—C13	−0.2 (3)
C8—C2—C3—C14	167.43 (16)	C11—C12—C13—C8	1.0 (3)
C2—C3—C4—O1	159.7 (2)	C9—C8—C13—C12	−0.9 (3)
C14—C3—C4—O1	−78.2 (3)	C2—C8—C13—C12	179.36 (17)
C2—C3—C4—C5	−19.8 (2)	N1—C6—C16—C21	−119.97 (17)
C14—C3—C4—C5	102.2 (2)	C5—C6—C16—C21	115.94 (18)
O1—C4—C5—C6	153.2 (2)	N1—C6—C16—C17	63.0 (2)
C3—C4—C5—C6	−27.2 (2)	C5—C6—C16—C17	−61.1 (2)
N7—N1—C6—C16	72.0 (2)	C21—C16—C17—C18	1.0 (3)
C2—N1—C6—C16	−122.28 (17)	C6—C16—C17—C18	178.05 (17)
N7—N1—C6—C5	−163.49 (17)	C16—C17—C18—C19	0.0 (3)
C2—N1—C6—C5	2.3 (2)	C23—O4—C19—C20	1.0 (3)
C4—C5—C6—N1	36.3 (2)	C23—O4—C19—C18	−179.06 (16)
C4—C5—C6—C16	161.94 (15)	C17—C18—C19—O4	178.80 (17)
C2—N1—N7—O2	−171.53 (17)	C17—C18—C19—C20	−1.3 (3)
C6—N1—N7—O2	−4.7 (3)	O4—C19—C20—C21	−178.57 (18)
N1—C2—C8—C9	−112.31 (19)	C18—C19—C20—C21	1.5 (3)
C3—C2—C8—C9	13.3 (3)	C19—C20—C21—C16	−0.5 (3)
N1—C2—C8—C13	67.46 (19)	C17—C16—C21—C20	−0.7 (3)
C3—C2—C8—C13	−166.89 (16)	C6—C16—C21—C20	−177.86 (17)
C13—C8—C9—C10	0.0 (3)	C4—C3—C14—C15	75.9 (3)
C2—C8—C9—C10	179.80 (17)	C2—C3—C14—C15	−161.5 (2)

### Hydrogen-bond geometry (Å, °)

**Table:**

*D*—H···*A*	*D*—H	H···*A*	*D*···*A*	*D*—H···*A*
C15—H15C···O1	0.96	2.56	3.163 (4)	121
C18—H18···O1^i^	0.93	2.56	3.472 (3)	167
C23—H23B···Cg1^ii^	0.96	2.89	3.718 (2)	144

Symmetry codes:  (i) −*x*+5/2, −*y*, *z*+1/2; (ii) *x*−1/2, −*y*+1/2, −*z*+1.
